# Themes and variations on piRNA-guided transposon control

**DOI:** 10.1186/s13100-023-00298-2

**Published:** 2023-09-02

**Authors:** Zuzana Loubalova, Parthena Konstantinidou, Astrid D. Haase

**Affiliations:** National Institutes of Diabetes and Digestive and Kidney Diseases, National Institutes of Health, Bethesda, MD USA

## Abstract

PIWI-interacting RNAs (piRNAs) are responsible for preventing the movement of transposable elements in germ cells and protect the integrity of germline genomes. In this review, we examine the common elements of piRNA-guided silencing as well as the differences observed between species. We have categorized the mechanisms of piRNA biogenesis and function into modules. Individual PIWI proteins combine these modules in various ways to produce unique PIWI-piRNA pathways, which nevertheless possess the ability to perform conserved functions. This modular model incorporates conserved core mechanisms and accommodates variable co-factors. Adaptability is a hallmark of this RNA-based immune system. We believe that considering the differences in germ cell biology and resident transposons in different organisms is essential for placing the variations observed in piRNA biology into context, while still highlighting the conserved themes that underpin this process.

## Background

The genomes of our germ cells live on in our children. Changes to the sequence of germline genomes impact the genetic make-up of future generations and the identity of a species. Genomes are vulnerable to threats by mobile genetic elements (transposons) that move or copy themselves into novel genomic locations [1–5]. Germline genomes are a battleground for genomic real estate and transposons have successfully taken possession of about half of our genomic space [3, 6]. Throughout evolutionary time, transposons have caused deleterious damage and expanded genome size [7–9]. They also provided a wealth of novel protein-coding and non-coding sequences [10–13]. Purifying selection eliminated deleterious insertions from the genetic pool and enriched for advantageous changes, and -in rare instances- transposon-derived sequences resulted in evolutionary innovation [14]. However, transposon activity results in DNA damage and mutagenesis, and novel insertions provide sequences for non-allelic homologous recombination. Therefore, germ cells developed protective mechanisms to minimize the deleterious effects of mobile genetic elements [15]. From bacteria to human, RNA-guided pathways provide adaptive defense against genome invaders. Animal germ cells use a specialized small RNA silencing pathway -PIWI proteins and their PIWI-interacting RNAs (piRNAs)- to silence resident transposons [16–18]. PiRNAs are one of three conserved classes of small silencing RNAs in eukaryotes [19]. In contrast to small interfering RNAs (siRNAs) and microRNAs (miRNAs) that associate with the AGO subfamily of Argonaute proteins, piRNAs associate with the PIWI subfamily. PIWI proteins are mostly expressed in germ cells, and loss of function mutations result in sterility [20]. PIWI-piRNA complexes recognize transposon transcripts by sequence complementarity in the nucleus and in the cytoplasm. Nuclear PIWI-piRNA complexes establish lasting epigenetic restriction that is faithfully maintained throughout embryonic development and adulthood. Cytoplasmic PIWI-piRNA complexes induce degradation of transposon transcripts and provide an acute response to active transposons. Here, we review conserved mechanisms of piRNA-guided restriction of transposon mobility from flies to human, elaborate on compelling variations, and establish context to transposon and germ cell biology.

### Conserved mechanisms of piRNA biogenesis and function, and their species-specific adaptations

**Fig. 1:**
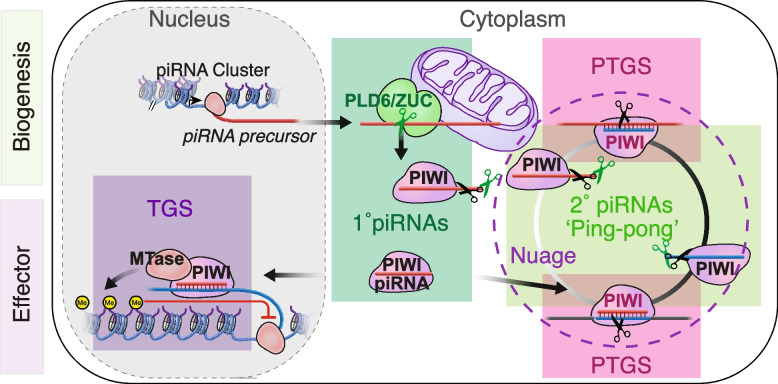
The basics of piRNA biogenesis and function in flies and mice. PiRNA precursors are transcribed form genomic regions called piRNA clusters. Long single-stranded RNA precursors are exported into the cytoplasm and transported to piRNA processing sites on the surface of mitochondria and in germ granules (also called ‘intermitochondrial cement’ or ‘nuage’). Primary piRNA biogenesis is initiated by the endonuclease D.m. Zucchini (ZUC)/ M.m. PLD6/MitoPLD on the mitochondrial surface (Biogenesis module 1). An individual long precursor gives rise to multiple piRNAs. PLD6/ZUC generates 5’phosphorylated RNA fragments that are loaded into PIWI proteins to form pre-piRNA complexes. 3’ end processing involves another endonucleolytic cleavage by PLD6/ZUC followed by exonucleolytic trimming in some organisms. During the effector phase of piRNA-guided silencing, mature PIWI-piRNA silencing complexes (piRISC) target RNAs with base-pairing complementarity. Nuclear PIWI-piRNA complexes recruit histone and DNA methyltransferases, depending on the organism, to establish transcriptional gene silencing (TGS) at target loci (silencing module 2). Cytoplasmic PIWI-piRNA complexes induce post-transcriptional gene silencing (PTGS) using PIWI’s intrinsic nuclease activity (slicer) (silencing module 1). Slicing of target RNAs produces 5’ monophosphorylated fragments that can either be further degraded by exonucleases or loaded onto a PIWI protein to generate secondary piRNAs (biogenesis module 2). Secondary piRNAs can induce further amplification of a piRNA-pair via coordinated slicing during ping-pong. (D.m … Drosophila melanogaster; M.m … Mus musculus)

PIWI proteins, the ZUC-processor nuclease (ZUC/PLD6), and the RNA helicases Vasa/DDX4 and Armi/MOV10L1 are conserved core-components of piRNA pathways and have long been known as essential germ cell factors [21]. Co-factors and regulators of piRNA biogenesis and function are largely species-specific. How does the essential piRNA pathway accommodate conserved traits while allowing for fast evolving adaptations? PiRNA pathways can be divided into different molecular modules with conserved core mechanisms (Fig. 1). During piRNA biogenesis, single-stranded RNA precursors are fragmented into small RNAs and loaded onto PIWI proteins to form functional PIWI-piRNA complexes. The first RNA cut is either performed by the endonuclease Zucchini/PLD6 (ZUC) (primary piRNAs) or by piRNA-guided slicing, which uses the nuclease activity of PIWI proteins themselves (secondary piRNAs or ‘ping-pong’) [22–25]. Either processing event generates RNA fragments harboring 5’ monophosphates, a prerequisite for association with PIWI proteins. After association with PIWI proteins, pre-piRNAs undergo 3’ end maturation to form mature PIWI-piRNA complexes [26]. During the effector phase of piRNA-mediated silencing, PIWI-piRNA complexes recognize target RNAs by complementary base-pairing and either recruit co-factors to establish transcriptional gene silencing (TGS) or induce post-transcriptional gene silencing (PTGS) through PIWI-mediated slicing and consecutive degradation of the target RNA [22, 27–30]. Individual PIWI proteins are loaded with primary or secondary piRNAs (or both), and either function in transcriptional or post-transcriptional silencing. The combination of biogenesis and effector modules varies for different PIWI proteins and provide opportunity for ‘mechanistic Lego’ during evolution.

PiRNA precursors are fast evolving and result in an everchanging repertoire of piRNA sequences [31, 32]. In flies and mammals, piRNA precursors are transcribed as long single stranded RNAs by RNA polymerase II. Other than that, little is known about the transcripts of transposon-targeting piRNA-precursors [33]. The genomic regions that encode these precursors are largely defined by mapping mature piRNAs to the reference genome and have been termed ‘piRNA clusters’ [22, 34]. In female germ cells of *Drosophila melanogaster*, a complex composed of the HP1 homolog rhino (rhi) and its co-factors deadlock (del) and cutoff (cuff) are required to initiate transcription and suppress splicing across dual-stranded piRNA clusters [35–39]. In adjacent ovarian somatic cells, a unidirectional piRNA cluster called Flamenco dominates piRNA production without requirement for rhi, del, or cuff. Flamenco (Flam) has long been identified as a major transposon control region and was later shown to be a prolific piRNA producing region [22, 40, 41]. Loss of Flam results in unleashed endogenous retroviruses, DNA damage, and female sterility [40, 42]. Until now, Flam remains one of only two piRNA clusters (the other being Su(Ste)) with a known phenotype that copies those of essential piRNA pathway genes including PIWI proteins themselves [32, 40, 42]. In male mice, piRNA-guided establishment of constitutive heterochromatin at young transposons occurs in embryonic gonads [27, 43, 44]. These pre-pachytene piRNAs originate from piRNA clusters and active transposable elements themselves [27]. Male germ cells operate a second wave of piRNAs during the pachytene stage of meiosis that remains mysterious in function but starts with well-defined piRNA precursors. Transposon sequences are under-represented in pachytene piRNAs, which enabled the precise mapping of transcription start and termination sites [45]. PiRNA generating regions might be as diverse as the piRNAs they produce, and we are just starting to identify determinants that mark individual transcripts for processing into piRNAs.

### The ZUC-processor complex generates primary piRNAs (PiRNA-biogenesis module 1)

The endonuclease Zucchini/PLD6 (ZUC) cleaves long single-stranded precursors into RNA fragments with 5’ monophosphorylated ends that can be loaded onto PIWI proteins to become primary pre-piRNAs. The ZUC/PLD6 nuclease is anchored into the outer mitochondrial membrane with its RNA-binding surface facing the cytoplasm. ZUC has been originally identified in a screen for female fertility in *Drosophila* and later shown to be essential for primary piRNA biogenesis in flies and mice [24, 25, 46–51]. ZUC/PLD6 belongs to an ancient family of HKD-phosphodiesterases that comprise phospholipases and nucleases [52]. Substrate specificity of these enzymes is determined by the shape of their substrate binding surface forming either a positively charged groove in case of nucleases or a potted structure for phospholipid head-groups [25]. The bacterial HKD-phosphodiesterase NUC cleaves double stranded DNA substrates in vitro [53]. In contrast, most eukaryotic HKD-enzymes act as phospholipases [52]. *Drosophila* ZUC and mouse PLD6 were the first eukaryotic HKD-enzymes to be characterized as nucleases [24]. Both enzymes are highly conserved in structure and function. Their substrate specificity is determined by a narrow substrate binding groove that can only accommodate single-stranded but not double stranded nucleotides. ZUC and PLD6 act as endonucleases, cleaving single-stranded RNA internally, and generate products with 5’ monophosphates and 3’ hydroxyl termini, a prerequisite for interaction with PIWI proteins. Additional processing preferences have been attributed to the ZUC processor complex, but whether they are cleavage preferences of the ZUC endonuclease or attributes of co-factors remains to be determined.

The preferences of primary piRNAs to harbor a uridine in the 5’ most position (1-U bias) is established in two steps during piRNA processing and PIWI-piRNA complex formation [54]. The ZUC-processor complex preferentially generates RNA fragments with a 5’ terminal pyrimidine (uridine or cytosine). Upon association with PIWI, cytosine is disfavored due to clashes with PIWI’s binding pocket [54]. Together, processing and PIWI-binding preferences establish the final 1U-bias. The preference for uridine immediately downstream of the cleavage position can also be observed at the 3’ end of *Drosophila* piRNAs and upon loss of the trimming exonuclease PNLDC1 in mice, and implicates the ZUC-processor in 3’end formation of piRNAs [55, 56]. If 3’ and 5’ ends of piRNAs are generated by a single ZUC cleavage event or associate with different ZUC-processing and loading complexes remains unknown.

### Secondary piRNAs and ‘ping-pong’ amplification of piRNA pairs (PiRNA-biogenesis module 2)

PIWI proteins are piRNA-guided nucleases that can cleave the target RNA across nucleotide 10 and 11 of the piRNA-guide [22, 23]. Their intrinsic ‘slicer’ activity is key for piRNA-guided post-transcriptional silencing (PTGS) in the cytoplasm, and mutations in key catalytic residues generate loss-of-function phenotypes [57, 58]. PiRNA-guided PIWI-nucleases generate RNA fragments with 5’ monophosphorylated termini that are either rapidly degraded by 5’ to 3’ exonucleases or loaded into another PIWI protein to become ‘secondary’ piRNAs [22, 23]. Loading of PIWI-generated RNA fragments into another PIWI protein requires the activity of the germline specific RNA helicase Vasa/DDX4 and is regulated by Tudor proteins [59, 60]. The newly generated PIWI-piRNA complexes can in turn slice an RNA target, and generate a 5’ monophosphorylated fragment that can be loaded into another PIWI protein, and so on. This coordinated slicing and piRNA production continues to amplify piRNA pairs and is known as the ‘ping-pong cycle’. ‘Ping-pong’ generation of secondary piRNAs can be directional and engage different PIWI proteins. Ping-pong in the ovary of *Drosophila melanogaster (D.m.)* is the prototype for ‘heterotypic ping-pong’ whereby Aubergine (Aub)-piRNA complexes generate slicing fragments that are loaded into Argonaute-3 (Ago3) and vice versa. Only Aub but not Ago3 can be loaded with primary piRNAs and initiates ping-pong. Ago3 can only be loaded with secondary (Aub-generated) piRNAs. Ago3-piRNA complexes generate secondary Aub-piRNAs and can induce further ‘phased’ piRNA production by the ZUC-processor complex [61, 62]. In contrast to heterotypic ping-pong in flies, mouse PIWIL2/MILI engages in ping-pong with itself called homotypic ping-pong [30]. Ping-pong is inhibited by the Tudor protein RNF17 during adult spermatogenesis in mice, and by lethal (3) malignant brain tumor (l(3)mbt) in *D.m.* ovarian somatic cells (OSC) [63, 64], and some *Drosophila* species do not operate ping-pong at all [65].

### PiRNA biogenesis occurs on the surface of mitochondria and in germ granules

ZUC/PLD6 resides on the surface of mitochondria. Its hydrophobic N-terminus is anchored in the outer mitochondrial membrane and its substrate binding surface faces the cytoplasm. Substrate binding and cleavage require a homodimer, and while little is known about the regulation of primary piRNA biogenesis, controlling dimer formation could be a key event. Secondary piRNA processing (ping-pong) is also linked to the surface of mitochondria and electron dense structures termed ‘inter-mitochondrial cement’. These dense, membrane-less RNA–protein compartments are called nuage (French for cloud) in *Drosophila* and germ granules (or ‘inter-mitochondrial cement’) in mammals [66]. The surface of mitochondria emerges as preferred location for innate immune response [67]. In vertebrates, innate immune sensors, among them the Dicer-related helicases Rig-I and MDA-5, also reside on the surface of mitochondria. The contribution of piRNA pathways to innate immune sensing in germ cells, and potential interactions with other innate immune sensors remains to be explored [68].

Contribution of membranes and electron-dense subcellular compartments has hampered the purification of piRNA processing complexes and identification of co-factors. Most co-factors have been identified in genetic screens for female fertility and transposon restriction in flies, and revealed a few conserved and many species-specific genes [46, 47, 69–71].

### Post-transcriptional gene silencing (PTGS) (Silencing module 1)

PiRNA-guided slicing of RNA targets requires the nuclease activity of PIWI proteins and induces target RNA degradation. Slicer activity has been originally observed for AGO-clade Argonaute proteins during RNA interference (RNAi) and later been attributed to an RNase H fold in the PIWI-domain of Argonaute proteins [72, 73]. SiRNA-guided slicing during RNA interference and piRNA-guided slicing are similar. However, the specific cellular and subcellular environment, intrinsic properties of PIWI-clade Argonaute proteins, and different co-factors influence the specificity and efficacy of piRNA-induced PTGS [74, 75]. In *Drosophila* ovaries*,* Aubergine (Aub)-associated piRNAs are mostly antisense to transposon sequences and guide slicing of complementary transposon transcripts [76]. In *Drosophila* testes, Aub-piRNAs derived from Suppressor of Stellate (Su(Ste)) induce degradation of the complementary Stellate transcripts, and loss of Su(Ste) or Aub results in male sterility [77–79]. In mouse testes, Piwil1/Miwi-piRNA complexes seem to induce slicing of mRNAs at low frequency without affecting the steady state level of these target RNAs [80]. The critical Miwi-piRNA targets required for male fertility remain elusive [57].

In the presence of the germ cell specific helicase Vasa/DDX4, piRNA-guided target cleavage can produce secondary piRNAs during ping-pong [59]. It remains unknown how frequent piRNA-guided slicing events result in the production of secondary piRNAs (see chapter on secondary (ping-pong) piRNA biogenesis, piRNA biogenesis module 2).

### Transcriptional gene silencing (TGS) (Silencing module 2)

PiRNAs establish lasting epigenetic silencing. Select PIWI-piRNA complexes translocate to the nucleus and mediate the de novo establishment of heterochromatin to inhibit transcription. Nuclear PIWI-piRNA complexes recognize nascent RNAs co-transcriptionally by base-pairing complementarity and recruit epigenetic modifiers. In flies, Piwi-piRNA complexes recruit the histone methyltransferase Eggless (Egg)/SETDB1 to establish trimethylation at Lysine 9 of Histone H3 (H3K9me3) and initiate transcriptional repression [81]. Efficient transcriptional repression of a target locus in flies has been linked to the combined abundance of complementary Piwi-piRNAs [82]. In mice, nuclear PIWIL4/MIWI2-piRNA complexes direct repressive histone modifications and de novo DNA methylation at target loci during early germ cell development and contribute to paternal imprinting [27, 44, 83, 84]. Loss of essential piRNA pathway genes results in overexpression of retrotransposons, DNA damage and meiotic arrest [60, 85–90].

### Different combinations of biogenesis and effector modules generate unique piRNA pathways for individual PIWI proteins

**Fig. 2:**
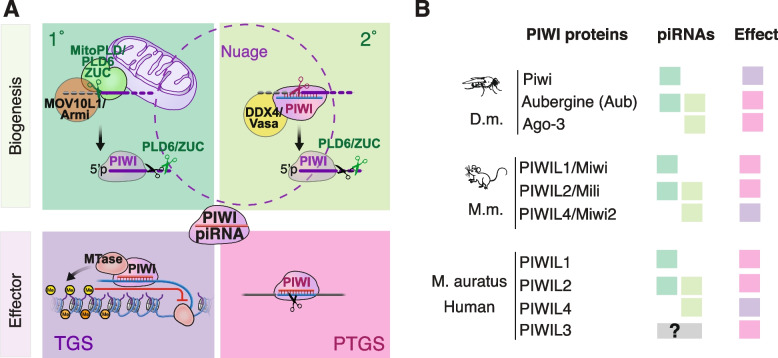
The combination of biogenesis and silencing modules differs for individual PIWI proteins in different organisms. **A** Primary (1°) piRNAs are generated by the endonuclease PLD6 (MitoPLD)/ Zucchini (ZUC) and its helicase partner MOV10L1/Armitage. A 5’ monophosporylated RNA fragment is loaded into a PIWI protein. The 3’ end of the mature piRNA is generated by another PLD6/ZUC cut, which is followed by exonucleolytic trimming in mice. Secondary (2°) piRNAs are generated by piRNA-guided slicing. The RNA helicase DDX4/Vasa partners with cytoplasmic PIWI slicers to load 5’ monophosphorylated cleavage fragments into another PIWI protein. During the effector phase, PIWI-piRNA complexes induce either transcriptional gene silencing (TGS) or post-transcriptional gene silencing (PTGS) depending on their subcellular localization and associated co-factors. **B** Individual PIWI proteins are either loaded with primary (dark green) or secondary (light green) piRNAs or both. Mature PIWI-piRNA complexes either induce TGS (purple) or PTGS (pink). (D.m. … Drosophila melanogaster; M.m. … Mus musculus; M. auratus … Mesocricetus auratus/ golden hamster)

The combination of biogenesis and effector modules differs for individual PIWI proteins and in different organisms (Fig. 2). PIWI proteins can be either loaded with primary or secondary piRNAs, or both, and function in either transcriptional or post-transcriptional silencing. In flies and mice, only one PIWI protein has the potential to transition to the nucleus when associated with piRNAs, the other PIWI-piRNA complexes remain in the cytoplasm and contribute to the post-transcriptional silencing. The RNA helicase Armitage/MOV10L1 associates with ZUC and is required for primary piRNA biogenesis in flies and mice [50, 51, 91–93] (Fig. 2A). The germ-cell specific helicase Vasa/DDX4 coordinates slicing and formation of secondary PIWI-piRNA complexes during ping-pong [59]. In analogy to the relationship of Dicer nucleases that partner with small double-stranded RNA binding proteins [94], piRNA processing nuclease seem to partner with RNA helicases. If and how this partnership contributes to substrate recognition or piRNA loading into PIWI proteins remains to be determined.

*Drosophila melanogaster* encodes three PIWI proteins: Piwi, Aubergine (Aub) and Argonaute-3 (Ago3) [16, 76] (Fig. 2B). Piwi associates with primary piRNAs and mature Piwi-piRNA complexes relocate to the nucleus to induce co-transcriptional silencing. Aub and Ago3 engage in heterotypic ping-pong initiated by Aub-piRNA complexes. Aub associates with primary and secondary piRNAs and cleaves target RNAs in the cytoplasm. Target-cleavage produces secondary piRNAs that are loaded into Ago3. Ago3 interacts with Aub-generated secondary piRNAs and cleaves target RNAs in the cytoplasm, which generates more ping-pong piRNAs that are loaded into Aub. Female germ cells express all three PIWI proteins, generate primary and secondary piRNAs and silence transposons at transcriptional and post-transcriptional levels.

The mouse genome encodes three PIWI proteins PIWIL1/MIWI, PIWIL2/MILI and PIWIL4/MIWI2 (Fig. 2B). PIWIL1/MIWI is loaded with primary piRNAs and degrades target transcripts in the cytoplasm. PIWIL2/MILI is loaded with primary and secondary piRNAs and cleaves target transcripts in the cytoplasm. PIWIL2/MILI engages in ping-pong with itself called ‘homotypic ping-pong’, and produces secondary piRNAs that are also loaded into PIWIL4/MIWI2. PIWIL4/MIWI2 interacts with MILI-produced secondary piRNAs, and mature PIWIL4/MIWI2-piRNA complexes translocate to the nucleus to establish co-transcriptional silencing at target loci. In contrast to mice, most other mammals encode a fourth PIWI protein, PIWIL3 that is specifically expressed in oocytes and associates with a mysterious class of short piRNAs [95–100].

### Differences in germ cell biology and endogenous mobile genetic elements change requirements for effective defense

Novel transposons and changes in germ cell biology require adaptations in piRNA-guided defense with new piRNAs and variable co-factors. In this chapter, we outline variations in piRNA pathways in the context of germ cell biology and the everchanging genomic landscape of resident transposons.

Different types of transposons invade germline genomes, adapt to distinct developmental stages and call for adaptations of the piRNA pathway for efficient defense [101–103]. In *Drosophila melanogaster*, the most active transposon family are gypsy endogenous retroviruses (ERVs) [104, 105]. These ERVs form infectious particles and threaten the genome integrity of neighboring cells [106–108]. To help protect germ cells from viral infection by neighboring cells, somatic cells of the germ cell niche operate a ‘Piwi-only’ piRNA pathway [109]. The somatic follicle cells of the *Drosophila* ovary express a single PIWI protein, Piwi, that is loaded with primary piRNAs and establishes co-transcriptional silencing of endogenous gypsy retroviruses. The restriction of gypsy elements in the ovarian somatic cells depends on the Flamenco piRNA cluster and is essential for germ cell health and fertility [22, 40, 42]. The presence of piRNA-guided transposon defense in the somatic cells of the gonad is specific to the *Drosophila* ovary and has not been observed in mammals.

Stage-specific adaptations by resident transposons result in variable phenotypes of piRNA pathway mutants. Adaptations of active transposons to specific developmental stages impact the phenotype of core piRNA pathway mutants. For example, loss of the RNA helicase MOV10L1 during mouse spermatogenesis results in unleashed transposons during meiosis [91, 93]. In golden hamster, the same loss-of-function allele manifests earlier during spermatogenesis and results in a failure to establish pre-meiotic spermatogonia [99, 100]. The difference in mutant phenotypes correlates with variations in resident transposons: spermatogonia formation in golden hamsters is associated with the activity of several transposon families, including the hamster-specific activity of the MYSERV element [99]. This observation suggests that the phenotypes of piRNA pathway mutants depend on the activity of resident transposons at different stages of development.

**Fig. 3:**
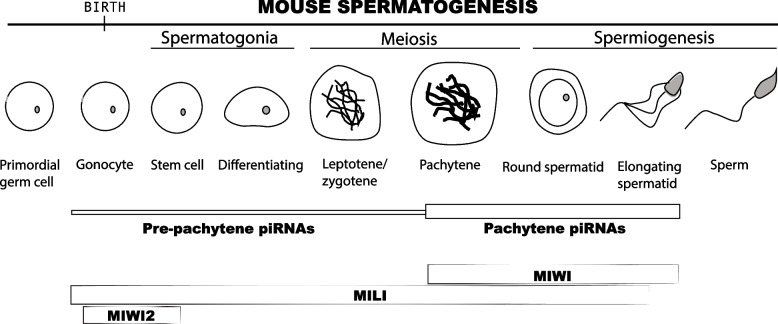
Expression of PIWI proteins and piRNA populations throughout mouse spermatogenesis. Mammalian germ cells express two distinct populations of piRNAs. Pre-pachytene piRNAs are mainly found in neonatal testis and persists at low levels until meiosis. Pre-pachytene piRNAs are associated with MILI and MIWI2 proteins, with MIWI2 being expressed mostly in embryonic gonads and shortly after birth, while MILI is expressed throughout most of the stages of spermatogenesis. Pachytene piRNAs are an extremely abundant population of small RNAs that begin expression during meiosis. They associate with MILI and MIWI proteins, with MIWI protein beginning expression at the same stage as pachytene piRNAs

Developmental timing separates two different classes of piRNAs in mammalian spermatogenesis (Fig. 3). Pre-pachytene piRNAs are expressed in pre-meiotic germ cells [30], while pachytene piRNAs begin their generation at the pachytene stage of meiosis I [34, 110]. Pre-pachytene piRNAs can be further divided into fetal and postnatal piRNAs. Fetal piRNAs associate with PIWIL2/MILI and PIWIL4/MIWI2 proteins and silence transposable elements [27, 30, 111]. PIWIL4/MIWI2 is the only mammalian PIWI protein that establishes lasting epigenetic restriction of transposons and participates in genomic imprinting [27, 43]. The expression of PIWIL4/MIWI2 is restricted to embryonic gonads and stem and progenitor spermatogenic germ cells [111]. Postnatal pre-pachytene piRNAs originate from transposons and mRNAs, and associate with cytoplasmic PIWIL2/MILI [30]. The third murine PIWI protein, PIWIL1/MIWI is not expressed until the pachytene stage of meiosis and interacts with pachytene piRNAs [112]. Transposon-derived sequences are under-represented in pachytene piRNAs, and ping-pong amplification is inhibited at this stage [63]. Pachytene piRNAs are produced from a little over one hundred long precursors that originate from mostly unannotated intergenic regions [45]. Knock-out of *Piwil1*/*Miwi* gene results in male sterility and germ cells arrest at the round spermatid stage [112]. The mutant phenotype depends on PIWIL1/MIWI’s ability to cleave target RNAs [57]. PIWIL1/MIWI-piRNAs show limited complementarity to transposons and protein coding genes and most seem to target little but their own region of origin. Two recent studies tested the biological significance of pachytene piRNA precursors, each of which produces thousands of piRNAs [113, 114]. Their results showed that piRNA precursors on chromosome 6 (pi6) and 18 (pi18) are required for the final steps of sperm maturation and the ability to fertilize eggs. The authors did not observe loss of transposon restriction or DNA damage, and their computational analyses suggest that individual piRNAs might regulate mRNAs instead. Loss-of-function of four other piRNA-generating regions remained inconsequential for fertility, and the mystery of pachytene piRNA-targets remains [114]. Neither pi6 nor pi18 phenotypes can explain the arrest of MIWI mutant sperm at round spermatid stage, and the key targets of pachytene piRNAs remain to be identified.

### Defining a new germline: preformation versus epigenesis [115–118]

The mammalian germline is induced *de-novo* from somatic cells. A signal from neighboring cells determines a small group of embryonic cells to become a primordial germ cell. Primordial germ cells proliferate and actively migrate to the developing gonads, where they commit to develop into an egg or sperm, based on the sex origin of the gonad [119, 120].

In contrast, the *Drosophila* germline is pre-formed by germ plasm that is composed of maternal determinates [121, 122]. Non-genetic memory can be contributed through germplasm to the germ cells of the next generation, and maternal piRNAs play an important role in germ cell specification and transposon control [123, 124]. If a new transposon is present in paternal genome but not in the maternal one, it mobilizes in the embryo and results in embryonic lethality. However, if the new transposon is present in the maternal but not the paternal genome, the mother contributes both the genomic transposon and piRNAs to protect the next generation. This phenomenon of hybrid dysgenesis can be observed for different transposons including P-element and I-element in flies. Introducing a new P-element either through the maternal (M strain) or paternal (P strain) germline resulted in different phenotypes [125, 126]. Introduction through the female germline resulted in healthy and fertile offspring, while introduction through the male germline generated sterile offspring. Sterility of dysgenic hybrids was associated with chromosome instability and mutations [127]. The different outcomes of genetically ident, reciprocal crosses suggested an underlying non-genetic component that was later identified as maternally contributed PIWI-piRNA complexes. These maternally contributed PIWI-piRNA complexes are major components of germ plasm and essential to protect the preformed germline of the offspring in flies [123, 128, 129].

### Differences in early embryogenesis

Activation of the zygotic genome occurs as early as the 2-cell stage in mice and hamsters, whereas it takes several rounds of division for this event to occur in flies [130]. While mouse and hamster had to adapt to a rapid process, fly can take an advantage of the cleavage period to regulate the maternal-to-zygote transition. In the fly embryo, piRNAs mediate the decay of *Nos* mRNA via a transposable element sequence in its 3' untranslated region (UTR) and thus impact patterning of the embryo [131]. PiRNA pathway mutants show no defects during embryogenesis in mouse and hamster. In contrast to the male-specific phenotype in mice, hamsters with mutations in key piRNA pathway genes exhibit male and female sterility. Oocytes devoid of *Mov10l1* or *Piwil*1 produce embryos that do not develop beyond the 2-cell stage. However, zygotes with homozygous deletions of *Mov10l1* or *Piwil*1 develop into adults. Thus, the developmental arrest of embryos from mutant oocytes is suggested to be a maternal effect [48, 91, 98–100, 111, 112, 132]. Exploring piRNA pathways across multiple model organisms is critical to identify both conserved and variable aspects of piRNA biology.

### Antiviral response and control over novel genomic invaders: RNA interference (RNAi) and piRNA pathways

**Fig. 4:**
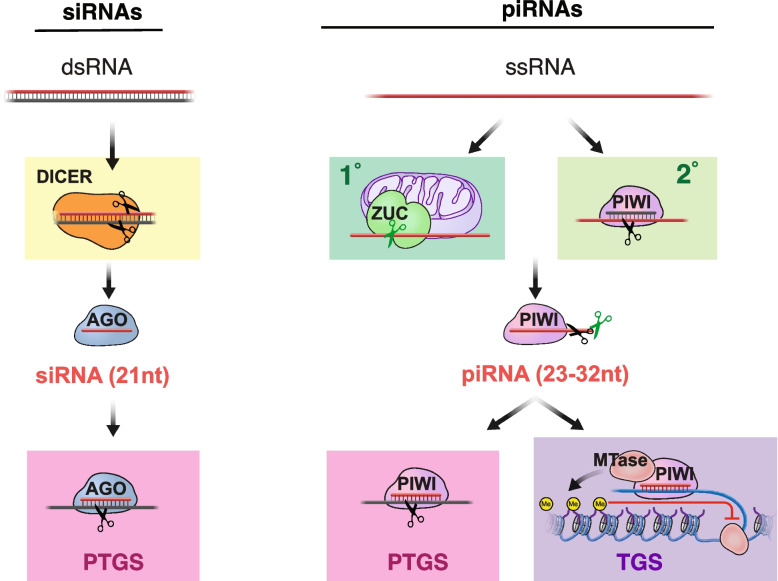
Similarities and differences between siRNA and piRNA pathways. Small interfering RNA** (**siRNA)-mediated RNAi pathway and piRNA pathway are ancient immune mechanisms against genome invaders. In both pathways, a small RNA associates with an Argonaute protein and mediates the sequence specific silencing of a target. SiRNAs associate with a member of the AGO subfamily of Argonaute proteins. PiRNAs are defined by their association with a PIWI-clade Argonaute protein. SiRNAs are defined by their biogenesis from long double stranded RNA (dsRNA) that is processed into small RNA duplexes by the RNase III enzyme Dicer. By contrast, fly and mouse piRNAs are generated from long, single stranded precursors in a Dicer-independent manner. Long single stranded RNAs (ssRNA) are processed by the conserved mitochondria-anchored endonuclease Zucchini (PLD6) or by the slicing activity of piRNA-guided PIWI proteins. SiRNAs guide the sequence-dependent slicing of a target RNA by their AGO protein partner, which exposes the target to exonucleolytic decay. Similarly, mature piRNAs mediate the slicing of complementary targets by their PIWI protein partner and induce target RNA decay. In addition to this post-transcriptional target regulation (PTGS), nuclear PIWI-piRNA complexes induce transcriptional gene silencing (TGS) by establishing restrictive epigenetic marks

The RNAi pathway is an ancient double-stranded (ds)RNA-sensing mechanism and the main anti-viral defense in fungi, plants and invertebrates [133–137]. Viral-derived dsRNA is processed into small interfering RNAs (siRNAs) by the RNase III enzyme Dicer [138] (Fig. 4). SiRNAs are loaded into AGO-clade Argonaute proteins to form mature RNA-induced silencing complexes (RISC) that identify target RNAs with extensive base-pairing complementarity [139, 140]. Using the slicer activity of the associated Argonaute protein, target RNAs are cleaved opposite of nucleotide 10 and 11 of the siRNA, and the generated 5’ monophosphorylated RNA fragments ensure rapid degradation by exonucleases [73]. The processing of viral dsRNA into siRNAs provides an innate and adaptive response to viral infection in plants and invertebrates that result in destruction of the viral RNA and generation of anti-sense siRNAs [133].

In mosquitos, viral infection induces siRNA and piRNA production [141], and virus-derived piRNAs were observed in testes of infected koalas [68]. Koalas are currently threatened by a retrovirus that establishes proviruses in germ cell genomes [142]. Some koala populations succumb to the virus, while others seem to have successfully gained control over the endogenized virus. Control over the endogenized virus correlates with antisense piRNAs in koala testes. Interestingly, viral transcripts triggered production of virus derived piRNAs in newly infected animal germ cells. Virus-derived piRNAs originated from the retroviral genome but not from its sub-genomic transcript. These ‘sense’ piRNAs resulted from degradation of the viral RNA and suggest an innate response by the piRNA pathway. Like the initial dicing of viral dsRNAs in invertebrates, breaking viral genomes into piRNAs might help to reduce the viral load and allow germ cells to survive until an adaptive defense can be established. With the emergence of the interferon response in vertebrates, innate immune sensors replace RNAi in dsRNA-mediated antiviral defense but piRNA pathways remained the major adaptive control over resident transposons and might also play a role in germ cell-specific innate defense [143].

### Mouse oocytes reactivate a functional RNAi pathway

While mammals generally lack an effective RNAi response, siRNAs are efficiently generated from long dsRNA by a specific Dicer isoform in mouse oocytes [144, 145]. This oocyte-specific Dicer isoform (Dicer(O)) is produced from an alternative promoter provided by a retrotransposon and is only expressed in murine oocytes. Dicer(O) lacks the N-terminal part of Dicer’s helicase domain that inhibits dsRNA processing and favors processing of pre-miRNA hairpins [146, 147]. In addition to viral dsRNA, gene-pseudogene pairs, convergent transcripts, and transposable elements provide an excellent substrate for Dicer cleavage in oocytes. Mouse oocytes produce siRNAs and piRNAs against transposons but only the siRNAs are essential and required for fertility [145, 148]. Dicer(O) deficient oocytes exhibit meiotic spindle defects and phenocopy the maternal *Dicer* null mutant [144, 148]. The meiotic defects preclude further investigation of potentially overlapping roles of piRNA and siRNAs at later stages of the female germline development.

SiRNA-dependent transposon restriction in mouse oocytes represent an exception to the rule. The importance of the piRNA pathway in the mammalian female germline has been demonstrated by recent studies in golden hamsters [98–100]. Results from these studies showed that mutations in key piRNA pathway genes result in female and male sterility, and described a function for PIWIL3-piRNA complexes for the first time. The oocyte specific PIWIL3 is conserved in other mammals including humans and suggests a universal role for piRNA pathways in female germ cell development [95–97, 149, 150]. However, since mice have been the primary mammalian model to study piRNA biology, mammalian reproductive biology focused on the essential function of piRNA pathways in male germ cells, and mutations in piRNA pathway genes have been correlated with male infertility [151]. Recent studies identified a novel class of piRNAs in human oocytes and provide a framework for addressing the importance of piRNAs for male and female fertility in humans [152].

## Summary and discussion

### Mechanistic ‘LEGO’: Flexible combinations of conserved piRNA biogenesis and silencing modules generate unique flavors of individual PIWI-piRNA pathways

PiRNA pathways integrate highly conserved and fast evolving genes and adapt to the ever-changing landscape of resident transposons [102]. While PIWI proteins, the nuclease ZUC/PLD6/MitoPLD, and key RNA helicases are conserved from flies to human, piRNA sequences, -precursors, and co-factors vary [16, 17, 19]. To integrate conserved mechanisms and species-specific variations, we propose a modular framework that broadly discriminates two processing and two silencing modules. First, piRNAs are generated from their long precursors by the endonuclease ZUC/PLD6/MitoPLD (primary piRNA biogenesis module) or by piRNA-guided slicing (secondary piRNA biogenesis module). Formation of functional PIWI-piRNA silencing complexes (piRISC) completes the piRNA biogenesis phase. Within piRISC, the sequence of the piRNA determines target specificity by complementary base-paring and the PIWI protein determines the fate of the targeted RNA resulting in either transcriptional or post-transcriptional silencing [16]. Cytoplasmic PIWI-piRNA complexes induce target-RNA degradation using PIWI’s intrinsic nuclease activity (post-transcriptional silencing module). Nuclear PIWI-piRNA complexes recruit histone and DNA methyltransferases -depending on the organisms- to establish heterochromatin formation and transcriptional silencing (silencing module 2). The modular architecture of piRNA pathways enables mechanistic ‘LEGO’: Individual PIWI proteins combine one or both biogenesis modules with a single silencing module, creating a unique flavor of their piRNA pathway.

The modular framework for piRNA biogenesis and function can be extended to other small RNA silencing pathways. Three classes of small silencing RNAs are conserved in eukaryotes: microRNAs (miRNAs), small interfering RNAs (siRNAs), and piRNAs [19]. MiRNAs and siRNAs are defined by their precursors and biogenesis mechanisms [153, 154]. MiRNAs are processed from RNA hairpin structures by the RNAse III enzymes Drosha and Dicer. SiRNAs are released from long dsRNA by Dicer. In contrast, piRNAs are defined solely by their association with PIWI-clade Argonaute proteins [34, 110, 155–157]. Historically, PIWI associated small RNAs required a new name in 2006, when the Zamore lab uncovered that PIWI-bound ‘repeat associated siRNAs (rasiRNAs)’ where not processed from long dsRNA and thus not siRNAs [155]. In the absence of understanding the precursors and biogenesis mechanisms of these small RNAs, the Hannon lab pragmatically renamed them ‘PIWI-interacting RNAs (piRNAs)’ [110]. The definition of piRNAs solely by their physical interaction with PIWI proteins was necessary, but bound to result in some confusion, and might warrant refinement in the future.

The discovery of ping-pong amplification of a primary piRNA trigger defined ‘secondary piRNAs’ [22, 23, 30]. ‘Primary piRNAs’ were later shown to be generated by ZUC/PLD6/MitoPLD [24, 25, 47, 48]. The ZUC-processor complex adds a novel biogenesis mechanism to small RNA biology by parsing long single-stranded RNAs into fragments with limited sequence specificity [24, 25, 54]. What determines and regulates primary piRNA biogenesis remains largely unknown [158].

In contrast to miRNAs, which exhibit a large degree of sequence conservation [153], piRNAs cannot be defined by sequence, and repositories of individual piRNA sequences are of limited value. Indeed, such databases have resulted in the inappropriate annotation of RNA fragments as ‘piRNAs’ in the absence of PIWI protein expression [159, 160].

Finally, additional piRNA biogenesis and effector modules have been identified in other organisms. C. elegans piRNAs (21U RNAs) are processed from individual short single-stranded RNA precursors by a novel processing mechanism [161, 162] and trigger 22G-RNA production in partnership with an RNA-dependent RNA polymerase [163]. Small RNA silencing pathways operate in all clades of life [136, 164, 165]. Their small RNAs are generated by a plethora of elegant processing mechanisms and RNA induced silencing complexes impact the fate of their target RNAs in different ways. They play essential roles in gene regulation and genome defense, and we are just beginning to understand the many flavors of small RNA silencing.

## Data Availability

N/A.
